# Application of artificial intelligence techniques for the profiling of visitors to tourist destinations

**DOI:** 10.3389/frai.2025.1632415

**Published:** 2025-08-04

**Authors:** Juan Schrader, Lloy Pinedo, Franz Vargas, Karla Martell, José Seijas-Díaz, Roger Rengifo-Amasifen, Rosa Cueto-Orbe, Cinthya Torres-Silva

**Affiliations:** ^1^Grupo de Investigación Innovación Turística y Comercio Exterior, Facultad de Ciencias Económicas, Administrativas y Contables, Universidad Nacional Autónoma de Alto Amazonas, Yurimaguas, Peru; ^2^Grupo de Investigación Transformación Digital Empresarial, Facultad de Ingeniería y Negocios, Universidad Privada Norbert Wiener, Lima, Peru; ^3^Grupo de Investigación Gestión ATEC, Facultad de Ciencias Económicas, Universidad Nacional de San Martín, Tarapoto, Peru

**Keywords:** artificial intelligence, segmentation, clustering, tourists, Agglomerative Clustering, DBSCAN, HDBSCAN, K-means

## Abstract

Tourism in Peru represents an opportunity for local development; however, there is limited understanding of visitor profiles. The aim of this study was to characterize tourists using machine learning techniques in order to identify distinct segments that can inform planning and promotional strategies for the Alto Amazonas destination. The research followed the CRISP-DM methodology for data analysis, based on surveys administered to 882 visitors. The data were processed using the clustering algorithms K-Means, DBSCAN, HDBSCAN, and Agglomerative, with Principal Component Analysis applied beforehand for dimensionality reduction. The results showed that the Agglomerative Clustering model achieved the best performance in internal validation metrics, allowing for the identification of five distinct visitor profiles. These segments provide valuable insights for the design of more inclusive and personalized tourism products. In conclusion, the study demonstrates the value of machine learning as a tool for tourism segmentation, offering empirical evidence that can strengthen the management of emerging destinations such as Alto Amazonas. The practical contribution of this study lies in providing strategic information that enables destination managers to tailor services and experiences to the characteristics of each segment, thereby optimizing visitor satisfaction and strengthening the destination’s competitiveness.

## 1 Introduction

Tourism has become one of the most dynamic industries worldwide, making a significant contribution to the economic development of many countries (Babafemi et al., 2023; Rebelo, 2022). Various authors highlight its capacity to generate income and employment, foster cultural exchange, and promote infrastructure development (Álava-García et al., 2024; Félix-Mendoza et al., 2024; León-Gómez et al., 2021). In this regard, it is recognized as an economic pillar that drives growth and sustainability in different communities, acting as a catalyst for progress (Pulido-Fernández and Cárdenas-García, 2021; Thommandru et al., 2023; Walker et al., 2021).

In the Peruvian context, tourism is highly significant due to its vast cultural, historical, and natural wealth (Riojas-Díaz et al., 2022; Zavaleta-Chavez-Arroyo et al., 2024). Machu Picchu stands out as one of the New Seven Wonders of the World, built by the Inca civilization in the 15th century and located high in the Andes Mountains. Likewise, the Nazca Lines are highlighted, which are pre-Columbian geoglyphs etched into the desert sands, covering nearly 1,000 square kilometers and comprising around 300 distinct figures, including animals and plants. Among Peru’s natural resources, the Gocta Waterfall, located in the Amazonas region, also stands out, ranking 17th among the tallest waterfalls in the world.

According to data from Ministry of Foreign Trade and Tourism (2024), between January and November of 2024, Peru received 2,976,151 international tourists. This statistic represents a 31.6% increase compared to the same period in 2023 and a 74.2% recovery relative to pre-pandemic levels in 2019. The most visited destinations by foreign tourists are located in the Cusco region, with the Historic Sanctuary of Machu Picchu leading the list with 981,666 visitors, followed by the Ollantaytambo Archaeological Park with 495,462; the Sacsayhuamán Esplanade with 439,139; the Moray Archaeological Complex with 393,988; and the Pisaq Archaeological Park with 327,877. However, despite its potential, there is a need for a better understanding of visitor characteristics knowledge that, according to Camacho Delgado et al. (2023), is essential for developing tourism promotion strategies, ensuring that destinations offer better experiences.

The problem identified lies in the lack of knowledge regarding the characteristics of visitors to Alto Amazonas, a Peruvian tourist destination that, due to its natural environment and cultural richness, offers a variety of tourism modalities, including ecotourism, cultural tourism, and recreational tourism. According to Martell-Alfaro et al. (2024) and Ruiz Camus et al. (2022), the main tourist attractions with the highest demand include Lake Cuipari, ideal for canoeing, artisanal fishing, and birdwatching; the community of Apangurayacu, known for its cultivation of Amazonian flowers; the Kumpanama petroglyphs, an archaeological site that remains largely unexplored; the San Lorenzo waterfall, where visitors can enjoy its cold waters; and the community of Canoapuerto, which offers immersive experiences related to the ancestral customs of the Indigenous Shawi people.

Lee and Kim (2023) mention that limited access to visitor information hinders decision-making by tourism managers, restricting their ability to attract and satisfy different tourist segments. Without a clear understanding of who the visitors are and what they seek, it becomes challenging to design offerings that meet their expectations or needs (Aksu et al., 2022). Among the causes of this problem, we identify that the collection and analysis of data on Alto Amazonas tourists have been insufficient and fragmented. Often, studies rely on surveys or traditional methods that fail to capture the complexity and dynamism of tourist behavior. Furthermore, according to Dolnicar (2022), low investment in emerging technologies for data analysis limits the ability to obtain strategic information about visitors.

Consequently, without an adequate characterization of tourists, destinations lose competitiveness (Nakhaeinejad et al., 2022). Additionally, tourist offerings may become irrelevant compared to the interests and needs of visitors, leading to decreased satisfaction and loyalty among tourists (Jarumaneerat, 2022). Moreover, the lack of information about visitor characteristics results in ineffective marketing strategies and poor management of tourism resources (Zhou and Chen, 2023).

In light of this reality, the use of artificial intelligence techniques, specifically machine learning, has proven to be an efficient solution (Egger, 2024; Marín Rodriguez et al., 2025; Penagos-Londoño et al., 2021; Yadegaridehkordi et al., 2021). According to Mor et al. (2023), this technique allows for the analysis of large volumes of data to uncover patterns and trends that would not be evident through traditional methods. Clustering, for example, can group tourists into segments based on their behaviors and preferences, thereby facilitating the creation of detailed and actionable profiles (Ghosh and Mukherjee, 2023; Tsegaw, 2023).

Although artificial intelligence has demonstrated increasing practical use in the tourism sector, its application in Amazonian regions remains limited. Most studies continue to rely on conventional approaches that fail to capture the complexity and heterogeneity of visitor behavior in these territories. Therefore, this study aims to apply machine learning techniques to characterize visitors to the Alto Amazonas tourist destination in Peru. By adopting this approach, we seek to identify distinct tourist segments to support the development of promotional and management strategies that enhance the visitor experience and foster sustainable tourism in the region.

## 2 Previous studies

In the tourism industry, segmentation is a widely addressed topic due to its relevance in generating information that supports the design of marketing and loyalty strategies. Among the reviewed studies, the work of Parra Vargas et al. (2021) stands out. They applied lifestyle-based segmentation to domestic tourists in Spain using a two-stage analysis that combined hierarchical and *k-means* clustering techniques. Based on demographic, personality, and lifestyle data, they identified four tourist profiles: Social, Activist, Cautious, and Adolescent. They also found significant differences in personality traits, highlighting the usefulness of psychographic segmentation for designing personalized tourism strategies.

In a different context, Nella and Christou (2021) segmented wine tourism in Greece using a multinational sample of visitors to 18 wineries. They applied statistical analyses such as chi-square and *t*-tests to identify significant differences between domestic and international visitors, as well as between first-time, repeat, individual, and group tourists. Their findings revealed differences in motivations, income levels, wine-related spending, brand loyalty, satisfaction, and post-visit attitudes. The authors demonstrate the value of a multidimensional segmentation approach for developing marketing strategies tailored to visitor profiles. From another perspective, Yao et al. (2021) conducted a segmentation of nautical tourism in China based on tourist motivations. Using survey data from visitors in Dalian, they applied a factorial analysis combined with clustering, identifying four main segments: novelty seeking, leisure and sport, multiple experiences, and self-fulfillment. The results offer deeper insights into tourist motivations and provide tourism operators with tools to design more effective management strategies.

We also highlight the study by Hassan et al. (2022), which focused on the segmentation of religious tourism in the city of Mecca based on pilgrims’ motivations. The authors applied a factorial and cluster analysis (*k*-means), identifying three motivational dimensions: religious, sociocultural, and shopping. Based on these dimensions, they identified three tourist segments: “Multiple Motivations,” “Passive,” and “Religious.” The first two segments showed high levels of satisfaction and loyalty toward the destination. Moreover, significant differences were found among the segments based on sociodemographic variables such as gender, age, education level, income, and number of visits. This study demonstrates the value of segmenting religious tourism by motivation to enhance the management and sustainability of sacred destinations.

In Latin America, several studies have explored the use of tourist segmentation techniques applied to local contexts through machine learning. Garcia Reinoso (2021) analyzed the segmentation of domestic tourism in Manta (Ecuador) based on tourist motivations, employing factorial techniques, cluster analysis, and dependency analysis. This study made it possible to identify differentiated tourist profiles and develop strategies based on the complementarity between sun-and-beach tourism and cultural heritage. Similarly, Penagos-Londoño et al. (2021) applied genetic algorithms and finite mixture models (latent class analysis) to segment tourists in Chile and Ecuador according to their perceptions of sustainability and destination trustworthiness. The study identified three segments (extremely optimistic, optimistic, and moderately optimistic), demonstrating that such perceptions can serve as useful criteria for managing destinations more sustainably.

In addition, Carvache-Franco et al. (2023) applied the K-means algorithm to segment urban tourists in cities across Mexico, Peru, Colombia, and Argentina, classifying them into three groups: those oriented toward multiple attractions, those focused on basic services, and passive tourists. This study demonstrates how unsupervised techniques can reveal emerging profiles in Latin American urban environments. Along the same lines, Carvache-Franco et al. (2024) analyzed visitors to protected areas in Ecuador using factorial analysis and hierarchical clustering, identifying three segments: basic recreation, landscape appreciation, and multiple-use recreation, with important implications for environmental and tourism planning. On the other hand, Pérez Gálvez et al. (2021) implemented multivariate segmentation techniques to analyze the profiles of tourists in natural parks in Colombia, incorporating attitudinal variables related to the environment, which enabled the classification of visitors into groups with different orientations toward sustainability.

As evidenced by the studies reviewed, segmentation contributes to a deeper understanding of tourist behavior by enabling the identification of differentiated profiles based on motivations, lifestyles, and other relevant criteria. This practice not only facilitates the development of effective marketing strategies but also supports the personalization of tourism offerings, enhancing the user experience and fostering loyalty. Therefore, segmentation should not be regarded merely as a commercial tool but also as a means to promote sustainability, innovation, and inclusion in tourism planning.

### 2.1 Tourist segmentation

Market segmentation is a field within consumer behavior studies that enables the division of a heterogeneous market into more homogeneous groups with similar characteristics, motivations, or behaviors. This technique, originally developed by Smith (1956) and later adapted to tourism by Mazanec in 1984, has become a key tool for improving planning, designing tourism products, and supporting strategic decision-making at destinations (Parra Vargas et al., 2021). Understanding the differences between segments allows tourism managers to tailor their offerings to the actual needs of visitors, thereby increasing satisfaction and fostering loyalty (Blanco-Moreno et al., 2024).

Traditionally, segmentation has been based on sociodemographic variables such as age, gender, educational level, or income. However, while these variables are useful, they are often insufficient to fully explain tourist decisions and preferences. As a result, psychographic segmentation techniques—which take into account aspects such as lifestyles, values, motivations, and personality—have gained prominence (Molina Collado et al., 2007). These dimensions provide a more comprehensive and predictive understanding of tourist behavior, especially in a context increasingly shaped by the demand for personalized experiences (Choe and Tou, 2025).

Within this framework, lifestyle segmentation has emerged as one of the most effective approaches within psychographic segmentation (Agarwal and Singh, 2021). This technique is based on identifying behavioral patterns related to activities, interests, and opinions, and has proven useful across various types of tourism, including urban, cultural, and nature-based tourism (Tasci et al., 2022). Moreover, recent studies, such as that of Parra Vargas et al. (2021), have incorporated personality traits as a complementary explanatory variable, demonstrating that this combination allows for more precise identification of tourist profiles.

Based on these premises, tourist segmentation is not only useful for destination marketing and promotion, but also for sustainable management and innovation within the sector. It enables the design of differentiated experiences, the anticipation of behavioral trends, and the ability to respond to the evolving expectations of travelers. Through more complex and integrative approaches—such as segmentation based on lifestyle and personality—it is possible to move toward smarter, more inclusive, and visitor-centered tourism planning.

### 2.2 Machine learning models for segmentation

Machine learning is a subfield of artificial intelligence focused on developing algorithms capable of learning from data and performing specific tasks without explicit programming (El Naqa and Murphy, 2015). Its main goal is to enable systems to identify complex patterns, make decisions, and generate predictions through the analysis of large volumes of data (Perales-Domínguez et al., 2024; Shinde and Shah, 2018). In the tourism sector, machine learning has gained increasing importance, particularly in processes such as service personalization, demand forecasting, and market segmentation (Bartra-Rategui et al., 2024; Núñez et al., 2024).

Within machine learning, segmentation is primarily carried out using clustering algorithms, an unsupervised learning technique that identifies natural groupings within data without requiring predefined labels (Al-Omary and Jamil, 2006). These methods are useful for dividing tourists into profiles or segments with similar characteristics, based on variables such as behavior, preferences, or lifestyle (Hassan et al., 2022). Unlike traditional techniques, clustering models can process complex, multidimensional datasets, offering a more accurate and nuanced understanding of target audiences (Huamán et al., 2022; Valles-Coral et al., 2022).

Among the most commonly used models is K-Means, a partitioning algorithm that groups data into *k* clusters defined by the user. Its simplicity and efficiency have made it a widely employed tool in tourism studies (Jauhari et al., 2022). On the other hand, hierarchical algorithms such as Agglomerative Clustering construct a tree-like clustering structure (dendrogram) by progressively merging similar observations, offering a visual representation that is useful for understanding relationships between segments (Rodríguez et al., 2018).

In addition, there are density-based clustering models such as DBSCAN (Density-Based Spatial Clustering of Applications with Noise) and HDBSCAN (Hierarchical DBSCAN), which can identify arbitrarily shaped clusters and detect noise or outliers (Pensiri et al., 2022). These techniques are particularly well-suited for tourism data that lack clearly defined structures or exhibit irregular distributions (Faizal et al., 2025). Their main advantage is that they do not require specifying the number of clusters in advance and are capable of capturing more realistic patterns in heterogeneous scenarios (Fuchs and Höpken, 2022).

Based on the literature, it can be stated that machine learning models offer new possibilities for tourism segmentation, overcoming the limitations of traditional techniques. By applying clustering algorithms, more accurate, flexible, and context-adaptable segments can be obtained. This enables the design of more personalized and effective strategies in both marketing and destination management, fostering tourism experiences that are better aligned with the real interests and behaviors of visitors.

## 3 Materials and methods

We adopted the CRISP-DM (CRoss-Industry Standard Process for Data Mining) methodology, which provides a structured and standardized approach for conducting data mining and machine learning projects (Gonçalves et al., 2023). This methodology is divided into six main phases, each of which is essential for the success of the project.

**Figure 1:**
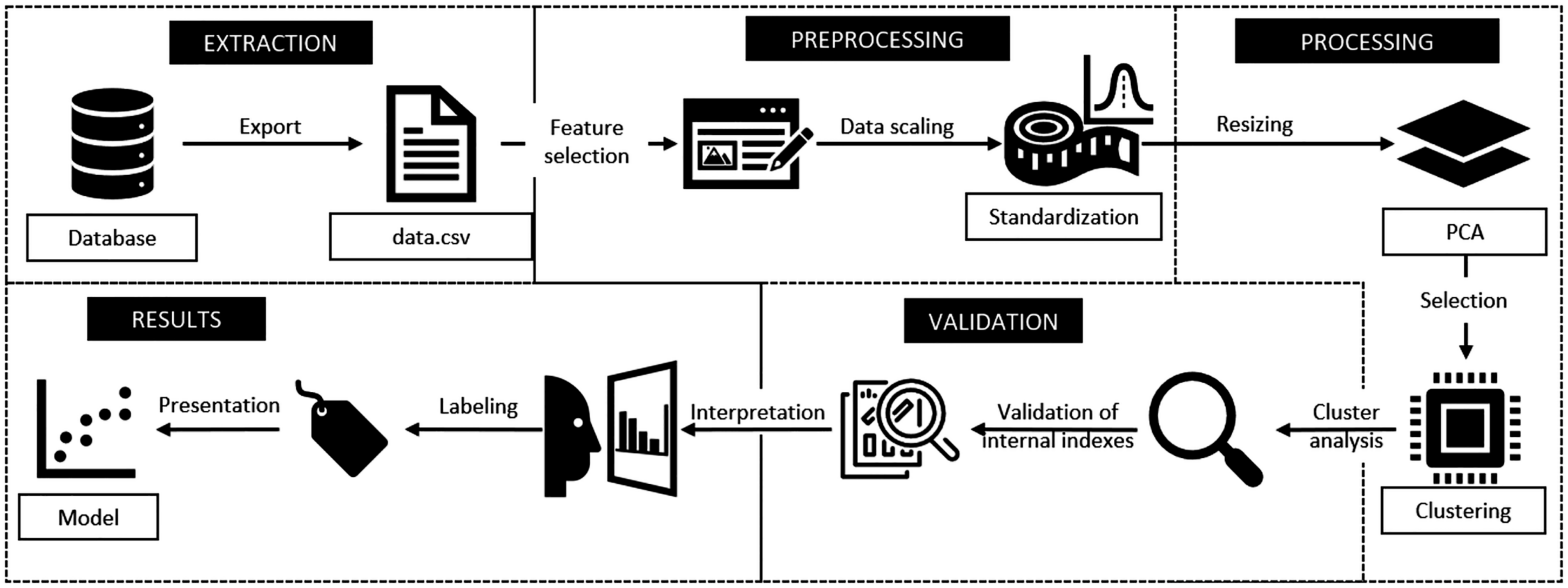
Technical workflow diagram aligned with CRISP-DM methodology.

Although the CRISP-DM methodology served as the overarching framework for this study, the following diagram (Figure 1) provides a complementary operational perspective of the technical workflow. It details the specific steps taken for data extraction, preprocessing, dimensionality reduction, clustering, and validation, thus illustrating the methodological flow from a data science implementation standpoint.

### 3.1 Business understanding

The province of Alto Amazonas, located in the western part of the Loreto region in Peru, has Yurimaguas as its capital city. Established on February 7, 1866, it borders the province of Datem del Marañón to the north and west, the provinces of Loreto and Requena to the east, and the San Martín region to the south. Covering an area of 18,764.32 km^2^, its territory ranges from mountainous areas along the border with San Martín to riverine lowlands on both banks of the Huallaga River, including sub-basins such as those of the Paranapura and Yanayacu rivers. The province is administratively divided into six districts: Yurimaguas, Balsapuerto, Jeberos, Lagunas, Santa Cruz, and Teniente César López Rojas. According to the 2017 census, it has a population of 122,725 inhabitants, making it the second most populous province in Loreto.

Its economy is primarily based on agriculture, extractive activities (forestry, hunting, and fishing), and commerce. The province is also recognized for its cultural diversity, being home to Indigenous communities such as the Shawis, Chayahuita, and Cocama, among others. Additionally, its strategic location in the Peruvian Amazon provides access to abundant natural resources, contributing to a growing influx of tourists. However, tourism stakeholders lack a clear understanding of visitor profiles, highlighting the need to identify and analyze their characteristics through homogeneous segments. This would support more effective promotion and management of the destination, better aligned with the needs and preferences of tourists. Therefore, this study aims to characterize visitors to the Alto Amazonas tourist destination using machine learning techniques.

### 3.2 Data understanding

For data collection, we used a survey technique through an *ad hoc* questionnaire specifically designed to characterize the profile of visitors to the Alto Amazonas province. The instrument included 38 variables, such as gender, age, occupation, income, among others, using categorical response scales. The surveys were administered both in person and virtually by the authors of this study, with the support of six previously trained university students. Surveys were conducted *in situ* on Fridays, Saturdays, and Sundays over a nine-month period, from August 2023 to April 2024, at the main tourist gathering points such as transport terminals, hotels, and the central square. The average response time was approximately 10 min. Prior to its field application, the instrument underwent a content validation process through expert judgment. It was presented to three academic specialists in tourism, who assessed each item based on the criteria of sufficiency, clarity, coherence, and relevance, using a dichotomous scale with “agree” and “disagree” options. This procedure ensured the content validity of the questionnaire.

Participants were selected using non-probability purposive sampling, reaching a total of 882 visitors to the province of Alto Amazonas. Only individuals over the age of 18 who stayed at least one night for tourism, recreational, academic, or commercial purposes were included. All participants voluntarily provided informed consent after receiving a clear explanation of the study’s objectives, thereby ensuring adherence to ethical principles of confidentiality, autonomy, and informed participation. The questionnaire is available upon request from the corresponding author.

### 3.3 Data preparation

The data preparation phase involved a systematic data engineering process to transform the raw survey responses into a format suitable for clustering algorithms. We prepared the data for analysis through a series of preprocessing steps in Python. The dataset initially consisted of 882 records corresponding to the responses provided by the surveyed participants. The process began with data cleaning, where duplicate records were removed. Missing values were handled using an imputation strategy: for categorical variables, the mode (most frequent value) of the respective column was used, while for numerical variables such as age, the mean was imputed. This process resulted in a dataset with 30 variables, which could pose a risk of multicollinearity and affect the performance of distance-based clustering algorithms.

Subsequently, feature engineering was performed. Categorical were converted into a numerical format using One-Hot Encoding. This technique creates new binary columns for each category, preventing the models from assuming an artificial ordinal relationship between categories. Finally, all numerical features, including the newly created binary ones and original numerical data like ‘Age’ and ‘Income’, were standardized using Standard Scaling (Z-score normalization). This step ensured that all variables contributed equally to the distance calculations in the clustering algorithms by transforming the data to have a mean of 0 and a standard deviation of 1. This entire process resulted in a fully numerical and normalized data matrix, ready for the application of Principal Component Analysis PCA.

**Figure 2:**
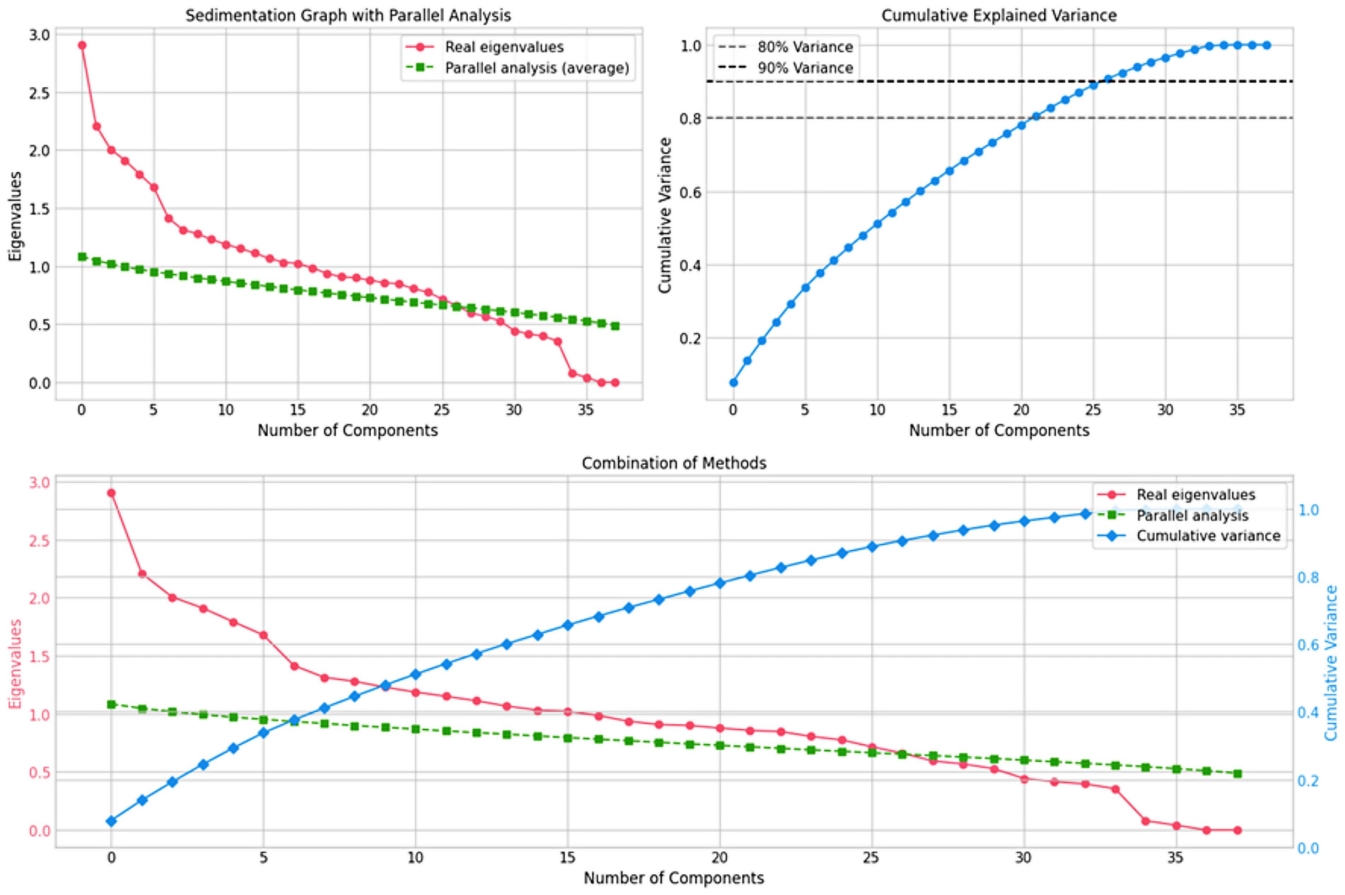
Determination of the optimal number of components using Parallel Analysis and cumulative explained variance. Source: Authors’ own elaboration.

The selection of the optimal number of components was determined using the Parallel Analysis method, a robust statistical technique that compares the eigenvalues of real data with those of random data generated with the same dimensions. The rule was adopted to retain all components whose true eigenvalue was greater than the mean eigenvalue of the Parallel Analysis. As shown in Figure 1, this criterion indicated that the number of statistically significant components to retain was 26. This choice was further supported by the fact that these 26 components explain approximately 90% of the total cumulative variance, ensuring a reduction in dimensionality without a significant loss of information for the subsequent cluster analysis (Zhang et al., 2025) (Figure 2).

### 3.4 Modeling

To gain a comprehensive understanding of tourist segments in Alto Amazonas, we selected a diverse set of clustering algorithms, each with distinct strengths. K-Means was included as a benchmark method widely used in tourism segmentation. Density-based algorithms, DBSCAN and HDBSCAN, were chosen for their ability to identify clusters of varying densities and effectively handle noise (Wang et al., 2022), characteristics often present in heterogeneous tourism data. Agglomerative Clustering was incorporated to explore potential hierarchical structures within the dataset (Wong et al., 2024). The purpose of this approach was to later evaluate and compare the performance of each technique to determine which yields the best results during the evaluation phase.

We began by using the Agglomerative Clustering algorithm with the preprocessed dataset, followed by the DBSCAN algorithm (Noorian Avval and Harounabadi, 2023), and the HDBSCAN algorithm (Wibowo et al., 2021) to identify clusters of different densities and effectively handle noisy data. Lastly, we applied the K-Means algorithm, which is widely used in numerous studies with satisfactory results (Wu and Yang, 2023).

### 3.5 Evaluation

We evaluated the quality of the clustering models using various internal validation metrics. We used the Silhouette coefficient to measure how well an element fits within the assigned cluster compared to other clusters, where a value close to 1 indicates that the points within a given cluster are cohesive and well-separated from other clusters (Rousseeuw, 1987):


$$S(i)=\frac{b(i)-a(i)}{\max\{a(i),b(i)\}\;\;}$$

where a(i)a(i)a(i) is the mean intra-cluster distance and b(i)b(i)b(i) is the mean distance to the nearest cluster for each sample.

We also used the Davies-Bouldin index, which evaluates the dispersion and separation of the clusters, where lower values indicate more compact and better-separated clusters.


$$\mathrm{DB}=\frac{1}{c}\sum_{i=1}^{c}\max_{i\neq j}\{\frac{d(X_{i})+d(X_{j})}{d(c_{i},c_{j})}\}$$

where *i* and *j* re the labeled clusters, $d(X_{i})$ and $d(X_{j})$ are the elements of those clusters *i* and *j* espectively, and $c_{i},c_{j}$ is the distance between the centroids of each cluster, *c* indicates the number of clusters.

Additionally, we applied the Calinski-Harabasz index, which considers the number of observations and the number of clusters, aiming to maximize the result as the number of clusters (k) changes. The index is calculated using the between-group sum of squares (BGSS) and the within-group sum of squares (WGSS), providing a measure of cluster separation (Calinski and Harabasz, 1974):


$$\mathrm{CH}=\frac{\frac{\mathrm{BSS}}{k-1}}{\frac{\mathrm{WSS}}{N-k}}$$

Where *N* is the total number of points.

These metrics allow us to objectively select the most effective clustering technique to characterize the profiles of Alto Amazonas visitors, focusing on the consistency of the groupings.

### 3.6 Deployment

For the deployment of the clustering model and the obtained information, it is proposed to design a cloud-based system in the future that allows tourism operators and local authorities in the Alto Amazonas province to access the analysis results easily and quickly. The clustering model should be integrated into an interactive platform that presents the different visitor segments and their characteristics through visual dashboards, using intuitive charts to display visitor behavior patterns and preferences, thereby facilitating data-driven decision-making.

## 4 Results and discussion

To begin, we performed hyperparameter tuning of the clustering algorithms using the Grid Search technique, based on maximizing the average Silhouette Coefficient. For the Agglomerative Clustering algorithm, we explored different hyperparameter configurations, such as the number of clusters (*n_clusters*), whose range was estimated to identify the expected segments or subsegments within the data. The linkage method and distance metric (*affinity*) were defined according to the available hyperparameters. The tested values were:

- n_clusters: [3, 4, 5, 6, 7, 8, 9, 10]
- linkage: [‘ward’, ‘complete’, ‘average’, ‘single’]
- affinity: [‘euclidean’, ‘l1’, ‘l2’, ‘manhattan’, ‘cosine’]

For the DBSCAN algorithm, we analyzed the hyperparameters *eps* (search radius), which was explored across a wide range based on the dispersion observed in the data, and *min_samples* (minimum number of samples), where lower values (3, 5) were selected to allow the identification of small groups, and higher values (7, 10) were tested to avoid excessive fragmentation of the data. The values considered were:

- eps: [0.3, 0.5, 0.7, 1.0, 1.5, 2.0]
- min_samples: [3, 5, 7, 10]

With HDBSCAN, being an algorithm that can find clusters of different densities, we adjusted the hyperparameters min_cluster_size (minimum cluster size), min_samples (minimum number of samples), and metric (distance metric). The tested configurations included:

- min_cluster_size: [5, 10, 15, 20]
- min_samples: [3, 5, 10]
- metric: [‘euclidean’, ‘manhattan’, ‘cosine’]

Finally, for the K-Means algorithm, we configured the hyperparameters *n_clusters*, which corresponds to the number of clusters to be identified, and *max_iter*, which determines the number of iterations the algorithm performs in a single run. We established values that ensure stability in cluster assignment without incurring excessive computation times:

- n_clusters: [3, 4, 5]
- max_iter: [300, 500]

After running and recording the various tests with the proposed configurations, the following results were collected as shown in Table 1.

**Table 1:** Internal validation.

Algorithm	Hyperparameters	Clusters	Silhouette	Davies-Bouldin	Calinski-Harabasz
Clustering Agglomerative	n_clusters = 3, linkage = single, affinity = ‘euclidean’	5	0.3593	0.4923	6.7046
DBSCAN	eps = 2.0, min_samples = 3	3	0.0453	2.3188	18.3665
HDBSCAN	min_cluster_size = 5, min_samples = 3, metric = ‘euclidean’	3	−0.0060	2.8605	48.7201
K-Means	n_clusters = 5, max_iter = 300	4	0.2039	1.4110	178.5731

Source: Authors’ own elaboration.

After evaluating the results provided by the various configurations, it was found that the clustering technique with the best results is Agglomerative Clustering, surpassing the other techniques in Silhouette and Davies-Bouldin coefficients, though it was only surpassed in the Calinski-Harabasz index by K-Means. Thus, Agglomerative Clustering is the best option according to the Silhouette metrics, which aligns with results obtained in similar contexts, such as Kingrat et al. (2023) who achieved a coefficient value of 0.405. It also aligns with the Davies-Bouldin coefficient, which, compared to the work of Ramadhani et al. (2023), shows improved results, indicating good consistency among the clusters obtained during this research.

**Figure 3:**
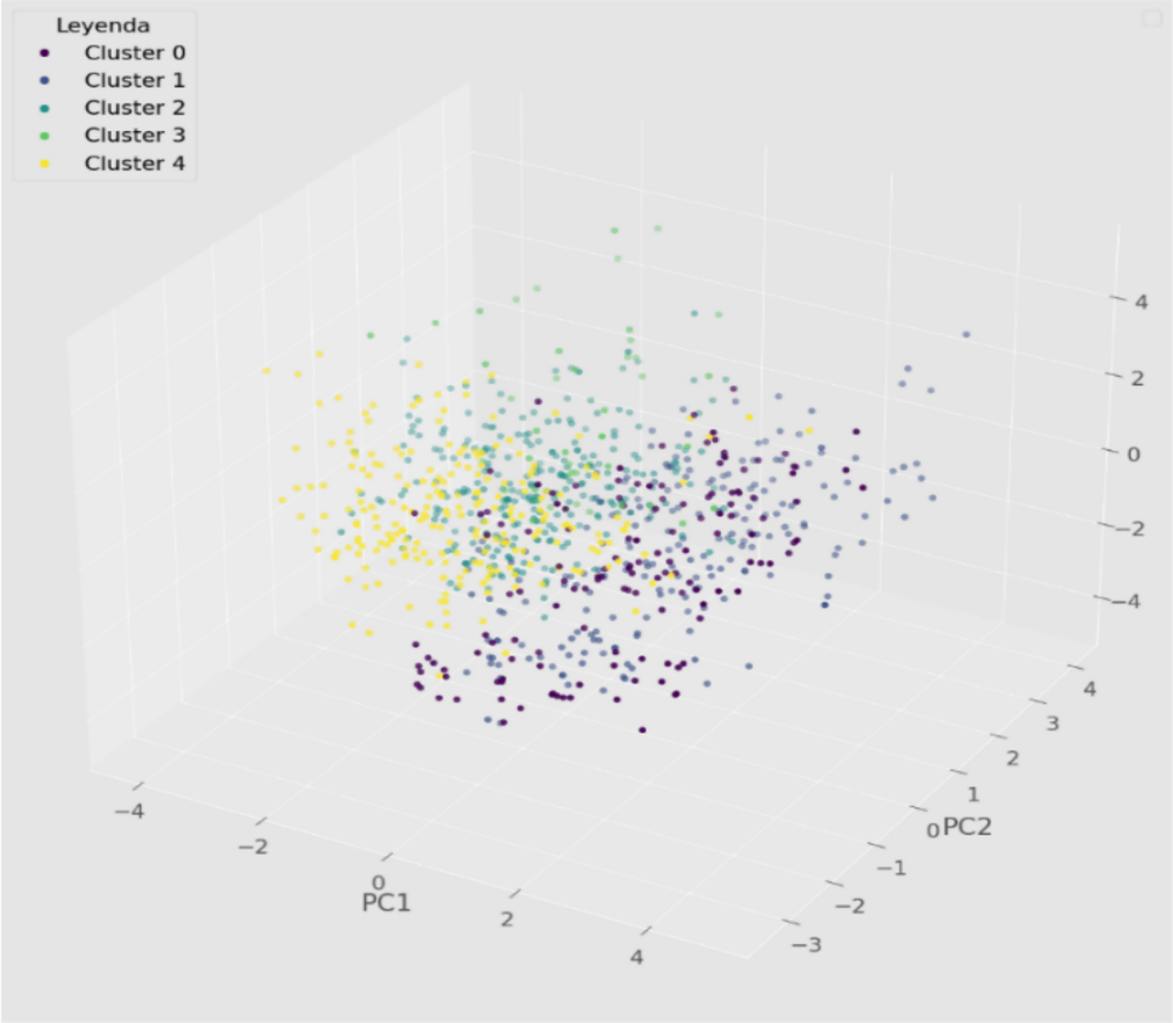
Three-dimensional graph of the distribution of clusters identified through Agglomerative Clustering. Source: Authors’ own elaboration.

The clustering analysis results reveal several distinctive segments among visitors to the Alto Amazonas province. We identified five main visitor groups, each with unique demographic, socioeconomic, and travel behavior characteristics (Figure 3).

**Figure 4:**
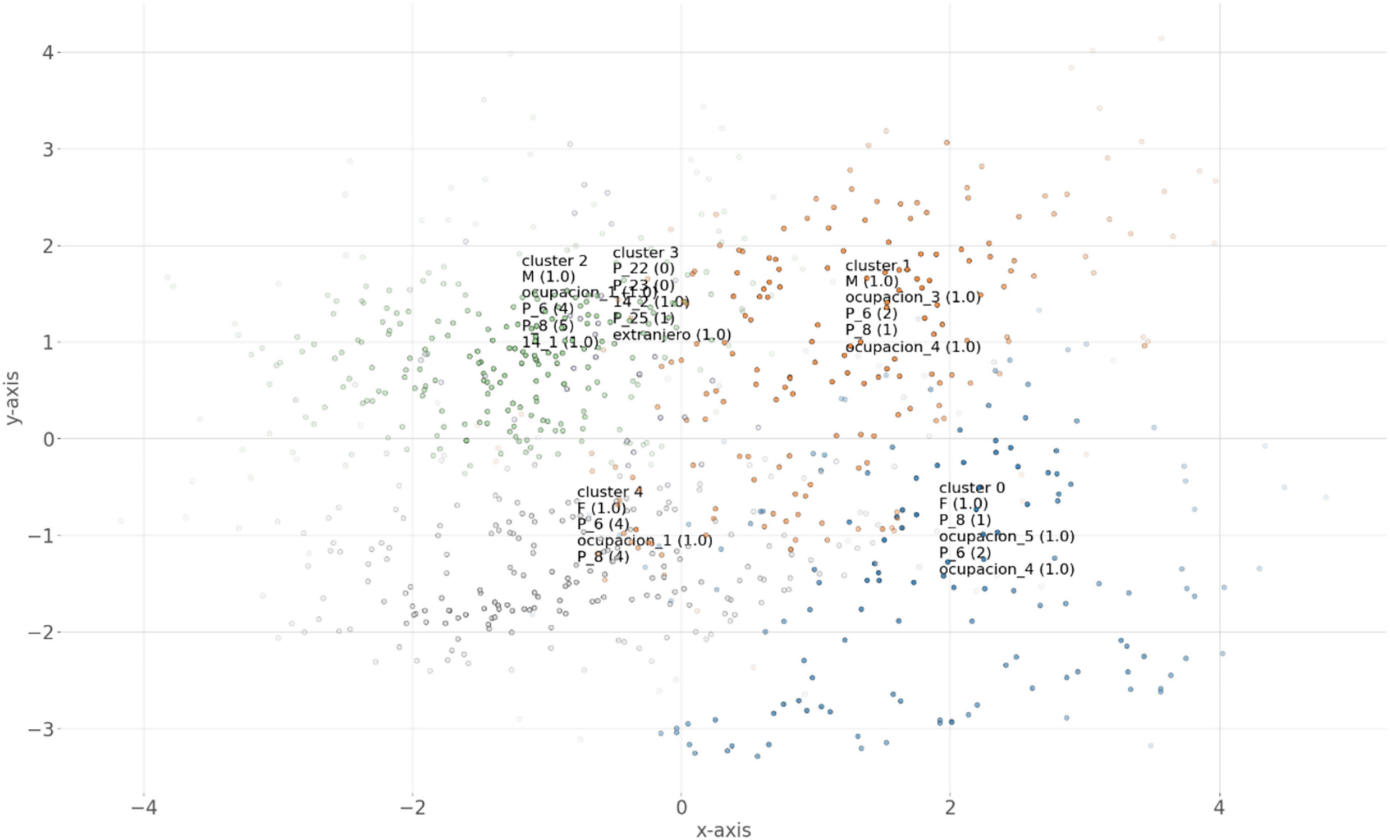
Visualization of the five visitor clusters with their dominant characteristics, generated using *enrichment* from hnet in clusteval. Source: Authors’ own elaboration.

The interpretation of the identified clusters was carried out through a visual analysis of the graph generated using the enrichment function from the hnet module, integrated into Python’s clusteval library. This tool allowed us to visualize the relationships between the distinctive features of each group in a two-dimensional space, where the proximity between points represents similarities in visitor profiles. Figure 4 illustrates the spatial distribution of the five clusters, with labels identifying the most relevant sociodemographic characteristics for each group, such as gender, educational level, income, among others.

Cluster 0 (C0)—Emerging Visitors with Local Affinity: This cluster is predominantly composed of women (*F*(1.0)) with monthly incomes below S/1,025.00 (P_8(1)). The most common educational level in this group is secondary education (P_6(2)), and their main occupations are homemakers (occupation_5(1.0)) and students (occupation_4(1.0)). In Figure 2, this cluster appears clearly defined in the lower right area, showing a significant spatial separation from the other groups. This segment represents an important target for the development of inclusive tourism in Alto Amazonas. Its socioeconomic profile, marked by limited income, highlights the need for economically accessible tourism offerings that enable participation in recreational and cultural activities. Despite financial constraints, this group constitutes a significant segment of visitors whose needs should be considered in destination planning efforts.

Cluster 1 (C1)—Flexible Visitors: This cluster is composed primarily of men (M(1.0)) with monthly incomes below S/1,025.00 (P_8(1)) and a secondary level of education (P_6(2)). Their predominant occupations are self-employed workers (occupation_3(1.0)) and students (occupation_4(1.0)). This segment shares similar economic characteristics with Cluster 0 but is clearly distinguished by the predominance of male participants and specific occupational roles. As shown in Figure 2, this group forms a well-defined segment in the upper right area of the two-dimensional space. Like Cluster 0, it represents a key target for the development of inclusive tourism aimed at visitors with limited budgets. Its occupational profile, combining self-employment and student status, suggests flexible time availability and a potential interest in activities that integrate learning and affordable recreation. Developing tourism offerings tailored to this segment would contribute to democratizing access to tourism in Alto Amazonas and generating a more consistent flow of visitors.

Cluster 2 (C2)—High-Income Visitor: This cluster is primarily composed of men (M(1.0)) with incomplete higher technical education (P_6(4)), who work in the public sector (occupation_1(1.0)) and earn monthly incomes exceeding S/4,100.00 (P_8(5)). This segment appears clearly differentiated in the upper left area of the graph (Figure 2), showing considerable distance from Clusters 0 and 1, which are characterized by lower income levels. It represents a significant opportunity for the development of higher value-added tourism services. Their greater purchasing power supports the implementation of higher quality standards in the local tourism offer, benefiting the sector as a whole. Additionally, their connection to the public sector could facilitate the development of institutional or corporate tourism, extending the tourism season beyond traditional vacation periods.

Cluster 3 (C3)—Discerning International Visitors: This cluster is predominantly composed of foreign visitors (foreign(1.0)) who report a low willingness to return (P_22(0)) or recommend the destination (P_23(0)), and who rate their experience negatively (P_25(1)). This segment appears in the central-upper area of the graph, clearly distinguishing itself from the other groups. Its position on the map (Figure 2) confirms it as a distinct profile with unique characteristics, particularly regarding its international origin and level of satisfaction with the tourism experience. This segment is especially relevant for the international projection of the destination. The low satisfaction expressed by this group serves as a significant warning regarding potential shortcomings in the tourism offer for international visitors. Enhancing this segment’s experience is essential for developing a sustainable inbound tourism strategy in Alto Amazonas and for improving the destination’s image in international markets.

Cluster 4 (C4)—Aspirational Visitors with Cultural Affinity: This cluster is predominantly composed of women (*F*(1.0)) with higher education (P_6(4)), who work in the public sector (occupation_1(1.0)) and earn monthly incomes between S/3,076.00 and S/4,100.00 (P_8(4)). This segment represents an opportunity to develop tourism with a stronger focus on cultural and educational aspects. Their educational level and moderate-to-high purchasing power suggest a potential interest in tourism experiences that combine learning, cultural authenticity, and quality services. Developing tailored offerings for this group could help diversify the visitor profile and enhance the destination’s cultural tourism offer.

Additionally, Table 2 presents the sociodemographic characteristics of visitors segmented through clustering techniques. Substantial differences emerge across clusters in terms of gender, age, and educational attainment. Cluster C0 primarily consists of young women with secondary education, while clusters C2 and C4 are composed mostly of visitors with university-level education. Cluster C3 stands out for its high proportion of older adults and international tourists, contrasting with the predominantly domestic profiles of the other groups. These distinctions reveal clearly differentiated segment identities, which are essential for designing targeted promotional strategies.

**Table 2:** Socio-demographic profile of each visitor cluster.

Variable	Indicator	C0	C1	C2	C3	C4
Gender	Female	81.40%	32.70%	19.30%	48.90%	74.10%
	Male	17.60%	62.80%	80.50%	45.70%	24.60%
	Other	1.00%	4.50%	0.20%	5.40%	1.30%
Age	<30 years	59.20%	38.90%	28.40%	18.60%	32.80%
	30–50 years	33.50%	49.10%	61.70%	52.30%	49.70%
	>50 years	7.30%	12.00%	9.90%	29.10%	17.50%
Source	National	99.50%	100.00%	100.00%	1.10%	100.00%
	Foreign	0.50%	0.00%	0.00%	98.90%	0.00%
Marital status	Single	51.20%	47.80%	22.50%	41.30%	29.80%
	Married	38.70%	44.90%	71.40%	47.60%	60.50%
	Divorced/Widowed/Other	10.10%	7.30%	6.10%	11.10%	9.70%
Education	Secondary or lower	79.40%	64.20%	9.80%	48.50%	11.20%
	Technician/Univ. inc.	17.80%	26.70%	59.60%	29.40%	32.70%
	Univ. full or more	2.80%	9.10%	30.60%	22.10%	56.10%

Source: Authors’ own elaboration.

Moreover, Table 3 reveals significant contrasts in socioeconomic characteristics. Cluster C0 comprises individuals with low income levels, mostly students and homemakers, whereas Cluster C2 includes high-income individuals, predominantly employed in the public sector. Cluster C4 represents a mid-range profile with medium income levels and greater occupational diversity. These socioeconomic variations suggest that income levels and employment status are closely associated with travel motivations and decisions, providing valuable insights for the development of differentiated tourism products.

**Table 3:** Socio-economic characteristics by cluster.

Variable	Indicator	C0	C1	C2	C3	C4
Revenue	<S/1,025	84.20%	69.80%	9.50%	28.30%	12.10%
	S/1,025–2,075	10.30%	22.60%	9.80%	29.70%	19.50%
	S/2,076–4,100	4.50%	6.40%	40.40%	24.60%	49.70%
	>S/4,100	1.00%	1.20%	40.30%	17.40%	18.70%
Occupation	Public sector	5.40%	9.70%	60.40%	9.80%	32.10%
	Private sector	5.80%	10.30%	9.40%	10.20%	10.30%
	Independent	11.30%	49.20%	11.50%	9.90%	9.80%
	Student	29.80%	19.40%	9.60%	10.50%	9.80%
	Housewife	40.70%	4.90%	2.30%	9.70%	5.50%
	Retired/not working	7.00%	6.50%	6.80%	49.90%	32.50%

Source: Authors’ own elaboration.

With regard to Table 4, it shows notable diversity in travel behavior. Clusters C2 and C4 are characterized by more frequent previous visits and longer stays, indicating a stronger connection to the destination. In contrast, clusters C0 and C1 reflect occasional travel patterns, short stays, and a preference for family or budget accommodations. Cluster C3, composed largely of international tourists, relies heavily on air travel and platforms such as Airbnb. This information is critical for designing loyalty strategies and diversifying accommodation and service offerings.

**Table 4:** Travel habits of visitor clusters.

Variable	Indicator	C0	C1	C2	C3	C4
Frequency of national tourism	Never	9.80%	10.20%	5.10%	19.70%	4.30%
	Rarely	59.60%	60.40%	15.20%	49.70%	20.20%
	Frequent	30.20%	29.40%	39.30%	19.80%	50.10%
	Very frequent	0.40%	0.00%	40.40%	10.80%	25.40%
Times visited Alto Amazonas	Once	79.30%	68.40%	18.70%	49.80%	38.60%
	≥2 times	20.70%	31.60%	68.90%	44.30%	61.40%
Overnight stay	1 night	88.90%	68.20%	29.30%	79.60%	38.50%
	2–3 nights	7.10%	20.30%	42.50%	14.20%	41.30%
	>3 nights	4.00%	11.50%	28.20%	6.20%	20.20%
Accommodation type	Hotel	48.50%	39.20%	28.60%	19.80%	25.70%
	Hostel	19.50%	24.70%	19.60%	14.70%	20.10%
	Shelter	9.70%	11.50%	15.20%	12.30%	10.40%
	Camping	4.80%	5.00%	9.40%	6.50%	10.20%
	Home of family or friends	12.80%	15.70%	15.00%	29.80%	28.60%
	AIRBNB	4.70%	4.00%	12.20%	16.90%	4.90%
Transport used	Private mobility	69.20%	64.80%	49.80%	39.10%	58.40%
	Loaned mobility	11.10%	15.30%	9.60%	9.90%	9.70%
	Travel agency	5.30%	5.70%	21.30%	10.40%	11.10%
	Airplane/Light aircraft	10.20%	9.80%	15.50%	29.70%	15.90%
	Other	4.20%	4.40%	3.80%	11.00%	4.90%

Source: Authors’ own elaboration.

As for Table 5, it shows that travel motivations vary considerably across clusters. While C0, C2, and C4 are primarily motivated by conventional tourism and cultural interests, Cluster C3 places greater emphasis on family reunions and affordability. Cluster C2 also includes a notable proportion of visitors driven by academic or research purposes, while C4 demonstrates a particular interest in traditional festivities and events. These findings highlight the specific destination attributes that appeal to each visitor segment.

**Table 5:** Primary travel motivations by cluster.

Variable	Indicator	C0	C1	C2	C3	C4
Travel motivations	Tourism	58.40%	49.70%	70.30%	29.90%	64.50%
	Commerce	9.80%	15.10%	6.20%	19.80%	9.70%
	Academic affairs	4.60%	4.70%	14.80%	10.10%	15.20%
	Family visit	10.30%	9.80%	5.20%	25.20%	9.80%
	Research	4.90%	8.80%	24.50%	14.70%	19.80%
	Scenic beauty	48.20%	44.80%	40.20%	19.80%	38.70%
	Economic prices	69.30%	64.40%	19.60%	28.40%	26.20%
	Traditional festivities (achiote carnival)	19.80%	24.70%	9.80%	24.60%	29.70%
	Other	4.50%	4.80%	9.60%	9.90%	6.40%

Source: Authors’ own elaboration.

Table 6 indicates that there are clear differences in culinary preferences, information sources, and preferred social media platforms. Cluster C0 highly values local cuisine and relies mainly on recommendations from relatives and friends. In contrast, C3 makes extensive use of digital channels and platforms such as YouTube and Airbnb. Clusters C2 and C4 combine various digital sources with a strong interest in regional gastronomy. These results underscore the importance of differentiated communication strategies aligned with the information-seeking behaviors of each group.

**Table 6:** Additional information sources and preferences.

Variable	Indicator	C0	C1	C2	C3	C4
Food preferences	Local gastronomy	79.10%	70.30%	60.20%	50.50%	65.40%
	Vegetarian gastronomy	11.20%	10.70%	15.30%	10.10%	10.50%
	Varied gastronomy	30.20%	35.40%	40.10%	29.70%	39.80%
	Fast food	22.90%	25.10%	10.40%	10.30%	15.20%
Information source	Internet	48.90%	60.20%	70.30%	80.10%	65.60%
	Television	12.30%	15.70%	9.80%	15.20%	10.40%
	Press	5.20%	5.40%	10.10%	10.30%	5.10%
	Tourism magazine	6.10%	5.90%	10.00%	10.40%	5.20%
	Friends and relatives	29.80%	25.30%	20.20%	15.10%	25.60%
	Tourism fairs	11.40%	10.70%	20.50%	10.30%	15.70%
	Radio	7.10%	5.80%	10.20%	10.50%	5.30%
	Other	6.90%	5.80%	5.70%	10.20%	5.40%
Social media platform	Facebook	69.30%	59.80%	50.20%	39.80%	54.70%
	WhatsApp	78.40%	75.60%	60.10%	49.90%	69.80%
	Twitter	20.50%	15.20%	10.00%	9.80%	15.30%
	YouTube	12.30%	10.40%	20.30%	20.20%	15.10%
	Instagram	60.70%	55.30%	50.00%	29.80%	59.90%
	Website	28.90%	25.60%	20.20%	15.00%	20.40%
Travel companion(s)	With friends	39.80%	45.20%	30.10%	20.30%	30.50%
	With your partner	31.20%	25.30%	20.40%	29.80%	25.10%
	With family	60.50%	49.70%	40.20%	29.90%	39.80%
	Coworkers	11.40%	15.10%	20.30%	10.40%	10.20%
	Classmates	21.60%	20.20%	15.00%	10.30%	15.30%
	Alone	9.70%	5.50%	10.10%	20.20%	10.10%

Source: Authors’ own elaboration.

Similarly, Table 7 shows that previous experiences, planning levels, and activities undertaken at the destination vary significantly. Cluster C0 has engaged with indigenous communities and frequently participates in nature-based activities. C2 and C4, with greater prior experience, tend to plan their trips in advance and diversify their activities. By contrast, C3 is characterized by limited knowledge of the destination and late-stage planning. These behavioral patterns reflect varying levels of connection to the territory and offer critical insights for enhancing the personalization of the tourist experience.

**Table 7:** Pre-trip behaviors and prior experiences.

Variable	Indicator	C0	C1	C2	C3	C4
District visited	Yurimaguas	69.30%	65.10%	40.20%	10.40%	30.50%
	Balsapuerto	12.10%	15.30%	20.10%	5.30%	20.20%
	Jeberos	5.80%	5.20%	10.40%	5.10%	10.30%
	Lagunas	5.40%	5.50%	10.30%	5.20%	10.10%
	Santa Cruz	5.70%	5.10%	10.20%	15.60%	10.00%
	Teniente César López Rojas	3.20%	3.10%	5.00%	15.30%	5.20%
	None	2.50%	0.70%	4.80%	53.10%	3.70%
Indigenous communities	Shawi	49.60%	40.20%	20.10%	10.30%	15.20%
	Achuar	10.80%	10.30%	10.00%	5.40%	10.50%
	Cocama–Cocamilla	10.30%	10.10%	20.20%	5.20%	10.30%
	Awajún	9.70%	15.20%	20.30%	10.10%	20.40%
	Candozi	5.20%	5.30%	10.10%	5.30%	10.00%
	Otro	4.60%	4.70%	4.80%	20.20%	9.70%
	None	8.70%	10.60%	10.20%	25.30%	20.10%
	Unaware of existence	1.10%	1.60%	5.40%	19.20%	4.80%
Tourist destinations visited	Cumpana	18.70%	25.20%	15.30%	10.10%	15.20%
	Lago Cuipari	50.90%	45.80%	40.20%	20.30%	40.50%
	Lago Sanango	32.10%	34.50%	25.00%	15.20%	30.10%
	Río Huallaga	40.30%	35.70%	30.30%	20.40%	35.40%
	Reserva Pacaya Sam.	25.50%	20.30%	30.10%	15.00%	25.30%
	Apangurayacu	29.70%	25.10%	20.20%	10.30%	20.20%
	Other	9.20%	9.20%	10.10%	15.10%	9.30%
Activities undertaken	Lake/lagoons/river walks	68.50%	64.90%	60.20%	40.40%	55.20%
	Walking in the countryside/nature areas	39.70%	45.10%	35.20%	25.30%	35.40%
	Go to restaurants	31.00%	35.30%	20.10%	25.20%	30.00%
	Walking in parks/plazas	21.20%	25.40%	15.00%	20.10%	25.30%
	See flora and fauna in their natural environment	49.90%	45.20%	40.50%	29.80%	45.10%
	Going to discotheques/karaoke/pubs	12.30%	10.20%	5.10%	15.20%	10.00%
	Buy handicrafts	19.80%	24.90%	15.00%	20.10%	20.20%
	Attending traditional festivals (achiote carnival)	14.90%	19.80%	9.90%	30.20%	25.10%
	Visiting native/native/peasant communities	24.00%	20.20%	15.30%	25.10%	20.30%
	Visiting nature reserves	19.80%	19.90%	20.20%	19.80%	19.80%
	Other, please indicate	6.90%	5.50%	5.40%	10.20%	6.40%
Trip planning horizon	Unplanned	39.40%	35.20%	20.10%	30.30%	25.10%
	≤1 week	31.20%	35.30%	40.00%	30.20%	35.20%
	2 weeks	11.30%	15.10%	15.00%	10.30%	15.30%
	3 weeks	5.20%	5.30%	10.10%	5.10%	10.40%
	1 month	8.80%	5.20%	10.00%	10.20%	10.10%
	2 months	3.70%	3.80%	2.90%	5.20%	3.70%
	>2 months	1.40%	1.60%	1.90%	19.50%	1.20%
Pre-trip information search	Yes	79.30%	75.10%	60.00%	49.90%	69.80%
	No	20.70%	24.90%	40.00%	50.10%	30.20%

Source: Authors’ own elaboration.

The findings of this study support the formulation of concrete intervention strategies aimed at strengthening tourism management in the Alto Amazonas destination. The segment of recurrent and well-informed visitors can be leveraged through loyalty programs, the creation of digital communities, and the design of exclusive tourism products that capitalize on their prior experience and intention to return. For tourists motivated by culture and gastronomy, and who possess higher educational levels, the development of thematic routes that integrate local festivals, cultural expressions, and traditional culinary experiences is proposed. Such offerings would not only diversify the tourism supply but also reinforce the destination’s identity and its competitive positioning.

In contrast, spontaneous visitors with limited budgets require targeted outreach strategies focused on immediacy, such as digital marketing campaigns and accessible basic services that facilitate quick travel decisions. The family-oriented segment demands improvements in hospitality infrastructure, safety measures, and recreational activities tailored to diverse age groups. Tourists with multiple motivations represent a hybrid profile that could be addressed through integrated packages combining nature, culture, and relaxation. These practical proposals, grounded in empirical and context-specific segmentation, provide essential inputs for public policy formulation, tourism product diversification, and institutional coordination for the sustainable development of Alto Amazonas.

It is important to note that visitor characteristics are unique to each tourist destination, as factors such as local culture, accessibility, specific attractions, and marketing draw different types of tourists (Coelho and Castillo Girón, 2023). Understanding visitor profiles allows for the adaptation of services and products to better meet their expectations; it also contributes to more efficient planning of promotional strategies and sustainable development of the destination (González Rosas et al., 2024).

The results show a methodological convergence with the findings of Carvache-Franco et al. (2024), who applied factorial analysis and hierarchical clustering to visitors of protected areas in Ecuador, identifying segments focused on basic recreation, landscape appreciation, and multiple motivations. Although both studies coincide in the diversity of tourist motivations and their utility for environmental planning, our research further incorporates the technological dimension and the role of prior information, offering a more comprehensive perspective on tourist behavior in rural settings.

The findings also align with the study by Carvache-Franco et al. (2023), who identified three urban tourist segments in cities across Mexico, Colombia, Peru, and Argentina using the K-means algorithm. While the urban context differs from the rural Amazonian setting, both studies revealed that sociodemographic variables—such as age, educational level, and income—play a key role in shaping visitor profiles. In our case, these variables not only influence the types of activities selected but also determine the sources of information used and the degree of trip planning. Unlike the urban context, the Alto Amazonas destination includes segments that are less familiar with digital platforms, posing challenges for designing adapted promotional strategies.

Furthermore, the attitudinal component explored by Pérez Gálvez et al. (2021) in natural parks in Colombia—using multivariate segmentation based on environmental attitudes—shows similarities with our results. Both studies highlight the importance of pro-environmental orientations in defining distinct tourist segments. However, our analysis introduces a broader array of motivational variables—such as price, social connections, gastronomy, and festivities—allowing sustainability to be interpreted not as a single criterion, but as part of a more complex system of decision-making. Similarly, the profiles identified by Penagos-Londoño et al. (2021) in Ecuador and Chile through finite mixture models and genetic algorithms reinforce the relevance of sustainability perceptions and destination trust as key segmentation axes, comparable to our more informed and recurrent visitor clusters.

In this regard, the study by Garcia Reinoso (2021) on domestic tourism in Manta (Ecuador), which used motivations, factorial analysis, and clustering techniques, supports the integration of motivational variables as a basis for segmentation. As in our research, a direct relationship was found between travel motives, behavior during the stay, and destination loyalty. However, our contribution goes further by employing artificial intelligence to model the data and incorporating variables related to digital channels, prior experience, and future intentions—thus expanding the methodological horizon of tourism studies in the Amazon region. Accordingly, the five segments identified not only offer actionable profiles for local destination management but also present a transferable model for other rural territories facing similar challenges in sustainability and strategic promotion.

This study represents a novel contribution to the field of tourism in emerging contexts, as it is one of the first to apply a comparative approach of clustering models—including K-Means, DBSCAN, HDBSCAN, and Agglomerative—to segment visitors in an Amazonian region of Peru. Unlike previous studies focused on consolidated or urban tourist destinations, our research provides empirical evidence on how advanced artificial intelligence techniques can uncover patterns in contexts where tourism data is scarce or fragmented. This approach not only expands the scientific understanding of tourism segmentation but also has a direct practical impact by providing actionable insights for local tourism managers facing data and resource constraints. In this sense, the study reinforces the potential of artificial intelligence as a democratizing tool for more inclusive and strategic tourism planning in rural and biodiverse regions such as Alto Amazonas.

It is important to acknowledge the methodological limitations of the present study, which should be considered when interpreting its findings. The target group analyzed corresponds specifically to visitors surveyed during weekends, which may not fully represent the total population of tourists throughout the week. This sampling decision was based on empirical evidence indicating that weekends concentrate the highest volume of tourist activity in the Alto Amazonas region, making them a critical window for analyzing behavioral patterns and segmentation. Nevertheless, this temporal delimitation restricts the generalizability of the results to other periods with potentially different visitor dynamics. Furthermore, the use of self-administered surveys may have introduced self-selection bias, as participation relied on the voluntary engagement of tourists, possibly influencing the composition of the identified clusters. These limitations should be taken into account in future research, which may consider complementary methodological approaches and broader temporal scopes according to the characteristics and operational objectives of each destination.

## 5 Conclusion

We successfully characterized the visitors to the Alto Amazonas tourist destination through the application of machine learning techniques. Using unsupervised clustering algorithms, we identified five visitor segments with distinct sociodemographic characteristics. The agglomerative clustering algorithm demonstrated the best performance in internal validation metrics, enabling the establishment of useful profiles for understanding the diversity of tourism in the region. These findings provide relevant insights into the different types of visitors to the destination, offering empirical evidence to support a more strategic approach to segmentation.

From a practical perspective, the results offer tourism stakeholders valuable information for decision-making in destination management, promotion, and planning. The characterization of segments allows for the adaptation of tourism products and services to the specific needs and expectations of each group, which can enhance the visitor experience, increase satisfaction levels, and promote loyalty. Moreover, identifying segments with lower levels of satisfaction, such as discerning international visitors, facilitates the design of corrective strategies to strengthen the destination’s global reputation. Altogether, these contributions support the development of a more competitive tourism sector in Alto Amazonas.

Moreover, the findings are not only relevant to tourism management in the Alto Amazonas destination, but also offer a replicable framework for other rural tourism contexts with similar characteristics. The application of unsupervised learning techniques enables the identification of distinct tourist profiles based on local data, which can be adapted to various territorial realities in Latin America and other regions seeking to enhance their tourism competitiveness through strategic segmentation. In this regard, the proposed model serves as a valuable tool for designing promotional and management policies grounded in evidence, contributing to both the sustainability and personalization of the visitor experience.

For future research, we recommend further exploring visitor motivations, perceptions, and emotions using mixed-method approaches that combine qualitative and quantitative techniques. Additionally, incorporating complementary data sources such as social media, digital reviews, or geolocation systems would enable a more dynamic understanding of tourist behavior. Finally, applying this methodology in other Amazonian or rural destinations in Peru would allow for the comparison of visitor profiles and the design of more integrated, evidence-based regional strategies.

## Data Availability

The raw data supporting the conclusions of this article will be made available by the authors without undue reservation.
